# A cost-sensitive online learning method for peptide identification

**DOI:** 10.1186/s12864-020-6693-y

**Published:** 2020-04-25

**Authors:** Xijun Liang, Zhonghang Xia, Ling Jian, Yongxiang Wang, Xinnan Niu, Andrew J. Link

**Affiliations:** 1 College of Science, China University of Petroleum, Changjiang West Road, Qingdao, 266580 China; 2 School of Engineering and Applied Science, Western Kentucky University, Bowling Green, 42101 KY USA; 3 School of Economics and Management, China University of Petroleum, Changjiang West Road, Qingdao, 266580 China; 4 Dept. of Pathology, Microbiology and Immunology, Vanderbilt University School of Medicine, Nashville, 37232 TN USA

**Keywords:** Peptide identification, Mass spectrometry, Classification, Support vector machines, Online learning

## Abstract

**Background:**

Post-database search is a key procedure in peptide identification with tandem mass spectrometry (MS/MS) strategies for refining peptide-spectrum matches (PSMs) generated by database search engines. Although many statistical and machine learning-based methods have been developed to improve the accuracy of peptide identification, the challenge remains on large-scale datasets and datasets with a distribution of unbalanced PSMs. A more efficient learning strategy is required for improving the accuracy of peptide identification on challenging datasets. While complex learning models have larger power of classification, they may cause overfitting problems and introduce computational complexity on large-scale datasets. Kernel methods map data from the sample space to high dimensional spaces where data relationships can be simplified for modeling.

**Results:**

In order to tackle the computational challenge of using the kernel-based learning model for practical peptide identification problems, we present an online learning algorithm, OLCS-Ranker, which iteratively feeds only one training sample into the learning model at each round, and, as a result, the memory requirement for computation is significantly reduced. Meanwhile, we propose a cost-sensitive learning model for OLCS-Ranker by using a larger loss of decoy PSMs than that of target PSMs in the loss function.

**Conclusions:**

The new model can reduce its false discovery rate on datasets with a distribution of unbalanced PSMs. Experimental studies show that OLCS-Ranker outperforms other methods in terms of accuracy and stability, especially on datasets with a distribution of unbalanced PSMs. Furthermore, OLCS-Ranker is 15–85 times faster than CRanker.

## Introduction

Tandem mass spectrometry (MS/MS)-based strategies are presently the method of choice for large-scale protein identification due to its high-throughput analysis of biological samples. With database sequence searching method, a huge number of peptide spectra generated from MS/MS experiments are routinely searched by using a search engine, such as SEQUEST, MASCOT or X!TANDEM, against theoretical fragmentation spectra derived from target databases or experimentally observed spectra for peptide-spectrum match (PSM). However, most of these PSMs are not correct [1]. A number of computational methods and error rate estimation procedures after database search have been proposed to improve the identification accuracy of target PSMs[2, 3].

Recently, advanced statistical and machine learning approaches have been studied for better identification accuracy in the post-database search. PeptideProphet [4] and Percolator [5] are two popular ones among those machine learning-based tools. PeptideProphet employs the expectation maximization method to compute the probabilities of correct and incorrect PSM, based on the assumption that the PSM data are drawn from a mixture of the Gaussian distribution and the Gamma distribution which generate samples of the correct and incorrect PSMs. Several works have extended the PeptideProphet method to improve its performance. Particularly, decoy PSMs were incorporated into a mixture probabilistic model in [6] at the estimation step of the expectation maximization. An adaptive method described in [7] iteratively learned a new discriminant function from the training set. Moreover, a Bayesian nonparametric (BNP) model was presented in [8] to replace the probabilistic distribution used in PeptideProphet for calculating the posterior probability. A similar BNP model [9] was also applied to MASCOT search results. Percolator starts the learning process with a small set of trusted correct PSMs and decoy PSMs, and it iteratively adjusts its learning model to fit the dataset. Percolator ranks the PSMs according to its confidence on them. Some works [10, 11] have also extended Percolator to deal with large-scale datasets.

In fact, Percolator is a typical method of supervised learning. With given knowledge (labeled data), supervised learning can train a model with labeled data and uses it to get an accurate prediction on unlabeled data. In [12], a fully supervised method is proposed to improve the performance of Percolator. Two types of discriminant functions, linear functions and two-layer neural networks, are compared. The two-layer neural networks is a nonlinear discriminant function which adds lots of parameters of hidden units. As expected, it achieves better identification performance than the model with linear discriminant function [12]. Besides, the work in [13] used a generative model, Deep Belief Networks, to improve the identification.

In supervised learning, kernel functions have been widely used to map data from the sample space to high dimensional spaces where data with non-linear relationships can be classified by linear models. With the kernel-based support vector machine (SVM), CRanker [14] has shown significantly better performance than linear models. Although kernel-based post-database searching approaches have improved the accuracy of peptide identification, two big challenges remain in practical implementation of kernel-based methods: (1) The performance of the algorithms degrades on the datasets with a distribution of unbalanced PSMs, in which case some datasets contain an extremely large proportion of false positives. We call them *“hard dataset”* as most post-database search methods degrade their performances on these datasets; (2) Scalability problems in both memory use and computational time are still barriers for kernel-based algorithms on large-scale datasets. Kernel-based batch learning algorithms need to load the entire kernel matrix into memory, and thus the memory requirement can be very intense during the training process.

In some extent, the above challenges also exists in other post-database searching methods. A number of recent works are related to the two challenges. The methods of data fusion [15–18] integrate different sources of auxiliary information, alleviated the challenge of “hard datasets”. Moreover, cloud computing platform is used in [19] to tackle the intense memory and computation requirement for mass spectrometry-based proteomics analysis using the Trans-Proteomic Pipeline (TPP). Existing researches either integrated extensive biological information or leveraged hardware support to overcome the challenges.

In this work, we develop an online classification algorithm to tackle the two challenges in kernel-based methods. For the challenge of “hard dataset”, we extend CRanker [14] model to a cost-sensitive Ranker (CS-Ranker) by using different loss functions for decoy and target PSMs respectively. The CS-Ranker model gives a larger penalty for wrongly selecting decoy PSMs than that for target PSMs, which reduces the model’s false discovery rate while increases its true positive rate. For the challenge of scalability problems, we design an online algorithm for CS-Ranker (OLCS-Ranker) which trains PSM data samples one by one and uses an active set to keep only those PSMs effective to the discriminant function. As a result, memory requirement and total training time can be dramatically reduced. Moreover, the training model is less prone to converging to poor local minima, avoiding extremely bad identification results.

In addition, we calibrate the quality of OLCS-Ranker outputs by using the entrapment sequences obtained from “Pfu” dataset published in [20]. Although the target-decoy strategy has become a mainstream method for the quality control in peptide identification, it cannot directly evaluate the false positive matches in identified PSMs. We aim to use the entrapment sequence method as an alternative of target-decoy strategy in the assessment of OLCS-Ranker [21, 22].

Experimental studies have shown that OLCS-Ranker not only outperformed Percolator and CRanker in terms of accuracy and stability, especially on hard datasets, but also reported evidently more target PSMs than those reported by Percolator on about half of datasets. Also, OLCS-Ranker is 15∼85 times faster on large datasets than the kernel-based baseline method, CRanker.

## Results

### Experimental setup

To evaluate the OLCS-Ranker algorithm, we used six LC/MS/MS datasets generated from a variety of biological and control protein samples and different mass spectrometers to minimize the bias caused by the sample, type of mass spectrometer, or mass spectrometry method. Specifically, the datasets include universal proteomics standard set (Ups1), the *S.cerevisiae* Gcn4 affinity-purified complex (Yeast), *S.cerevisiae* transcription complexes using the Tal08 minichromosome (Tal08 and Tal08-large) and Human Peripheral Blood Mononuclear Cells (PBMC datasets). There are two PBMC sample datasets which were analyzed with the LTQ-Orbitrap Velos with MiPS (Velos-mips) and MiPS-off (Velos-nomips) respectively. All PSMs were assigned by the SEQUEST search engine. Refer to [23] for the details of the sample preparation and LC/MS/MS analysis.

We converted the SEQUEST outputs from *.out format to Microsoft Excel format for OLCS-Ranker and removed all blank PSMs records if any. Statistics of the SEQUEST search results of the datasets are summarized in Table 1.

**Table 1:** Statistics of datasets

	Total	Target PSM	Decoy PSM
Yeast	14892	6703	8189
Ups1	17335	8974	8361
Tal08	18653	9907	8746
Tal08-large	69560	42222	27338
Velos-mips	301879	208765	93114
Velos-nomips	447350	307549	139801

A PSM record is represented by a vector of nine attributes: xcorr, deltacn, sprank, ions, hit mass, enzN, enzC, numProt, deltacnR. The first five attributes inherit from the SEQUEST algorithm and the last four attributes are defined as

- enzN: A boolean variable indicating whether the peptide is preceded by a tryptic site;
- enzC: A boolean variable indicating whether the peptide has a tryptic C-terminus;
- numProt: The number that the corresponding protein matches other PSMs;
- deltacnR: deltacn/xcorr.

Based on our observation, “xcorr” and “deltacn” played more important roles in identification of PSMs, and hence, we used 1.0 for the weights of the two features, and 0.5 for all others. Also, Gaussian kernel $k(x_{i},x_{j}) = \exp {(\frac {\|x_{i}-x_{j}\|^{2}}{2\sigma ^{2}})} $ was chosen in this experimental study.

The choice of parameters, *C*_1_,*C*_2_,*σ*, is a critical step in the use of OLCS-Ranker. We performed a 3-fold cross-validation and the values of parameters were chosen by maximizing the number of identified PSMs. Detailed cross-validation results could be found in Additional file 2. The PSMs were selected according to the calculated scores under FDR level 0.02 and 0.04, respectively, and FDR was computed using the following equation

$$\text{FDR}= 2D/ (D+T), $$

where *D* is the number of the spectra matched to decoy peptide sequences and *T* is the number of the PSMs matched to target peptide sequence. As the performance of OLCS-Ranker is not sensitive to the algorithm parameters, we constantly set *M*=1000, *m*=0.35|*S*|, where *S* is the active index set and |*S*| denotes its size, in this experimental study.

OLCS-Ranker was implemented with Matlab R2015b. The source code can be download from https://github.com/Isaac-QiXing/CRanker. All experiments were implemented on a PC with Intel Core E5-2640 CPU 2.40GHz and 24Gb RAM.

For comparison with PeptideProphet and Percolator, we followed the steps described in Trans Proteomic Pipeline (TPP) suite[24] and [10]. In PeptideProphet, we used the program MzXML2Search to extract the MS/MS spectra from the mzXML file, and the search outputs were converted to pep.XML format files with the TPP suite. In Percolator, we converted the SEQUEST outputs to a merged file in SQT format [25, 26], and then transformed it to PIN format by sqt2pin integrated in Percolator suite[10]. We used ’-N’ option of the “percolator” command to specify the number of training PSMs.

### Comparison with benchmark methods

We compared OLCS-Ranker, PeptideProphet and Percolator on the six datasets in term of the numbers of validated PSMs at FDR =0.02 and FDR =0.04. The performance of a validation approach is better if it can validate more target PSMs than the other approach under the same FDR. Table 2 shows the number of validated PSMs and the ratio of this number to the total of each dataset. As we can see, OLCS-Ranker identified more PSMs on three datasets, similar numbers of PSMs on the other three datasets, compared with PeptideProphet or Percolator.

**Table 2:** Number of PSMs output by PeptideProphet, Percolator, and OLCS-Ranker

Dataset	Method	FDR = 0.02			FDR = 0.04		
		Targets	Decoys	Ratio	Targets	Decoys	Ratio
Yeast	PepProphet	1379	13	0.206	1436	29	0.214
	Percolator	1225	12	0.183	1366	27	0.204
	OLCS-Ranker	1374	13	0.205	1467	29	0.219
Ups1	PepProphet	506	5	0.056	545	11	0.061
	Percolator	471	4	0.052	554	11	0.062
	OLCS-Ranker	473	4	0.053	528	10	0.059
Tal08	PepProphet	911	9	0.092	948	20	0.096
	Percolator	1036	10	0.105	1059	21	0.107
	OLCS-Ranker	1140	10	0.115	1156	22	0.117
Tal08-large	PepProphet	14966	152	0.354	15516	317	0.367
	Percolator	15793	159	0.374	16164	329	0.383
	OLCS-Ranker	15706	157	0.372	16078	327	0.381
Velos-mips	PepProphet	116533	1177	0.558	120080	2450	0.575
	Percolator	116046	1172	0.556	120952	2468	0.579
	OLCS-Ranker	117084	1182	0.561	120033	2448	0.575
Velos-nomips	PepProphet	166790	1684	0.542	173935	3549	0.566
	Percolator	165174	1668	0.537	174361	3558	0.567
	OLCS-Ranker	170722	1723	0.555	177007	3611	0.576

“Targets”: number of selected target PSMs; “Decoys”: number of selected decoy PSMs; “ratio”: the ratio of the number of selected target PSMs under FDR = 0.04 to the total number of target PSMs in the dataset; “PepProphet”: PeptideProphet

Compared with PeptideProphet, 25.1%, 4.9% and 2.4% more PSMs were identified by OLCS-Ranker at FDR =0.02 on Tal08, Tal08-large and Velos-nomips, respectively. Compared with Percolator, 12.2%, 10.0% and 3.4% more PSMs were identified by OLCS-Ranker at FDR =0.01 on Yeast, Tal08 and Velos-nomips, respectively. On Ups1 and Tal08-large OLCS-Ranker identified a similar number of PSMs to that of Percolator. The numbers of PSMs identified by the three methods on each dataset under FDR =0.04 are similar to those under FDR =0.02.

**Fig. 1:**
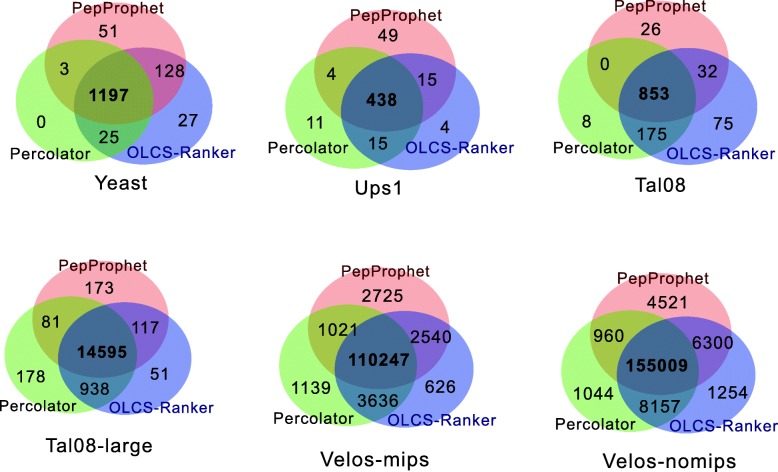
Overlap of identified target PSMs by PeptideProphet, Percolator and OLCS-Ranker. PepProphet: PeptideProphet

We have also compared the overlapping of target PSMs identified by the three approaches as a PSM reported by multiple methods is more likely to be correct. Figure 1 shows that the majority of validated PSMs by the three approaches overlaps, indicating high conference on the identified PSMs output by OLCS-Ranker. Particularly, on Yeast, the three approaches have 1197 PSMs in common, covers more than 86% of the total target PSMs identified by each of the algorithms. This ratio of common PSMs is 86% and 75% on Ups1 and Tal08, respectively, and more than 90% on Tal08-large, Velos-mips and Velos-nomips.

Furthermore, the overlapping PSMs identified from OLCS-Ranker and each of PeptideProphet and Percolator is more than those overlapping PSMs identified from PeptideProphet and Percolator. On Yeast, besides the overlapping among three methods, OLCS-Ranker and PeptideProphet identified 128 PSMs in common and OLCS-Ranker and Percolator identified 25 PSMs in common. In contrast, PeptideProphet and Percolator have only 3 PSMs in common. Similar patterns occurred on other datasets.

**Fig. 2:**
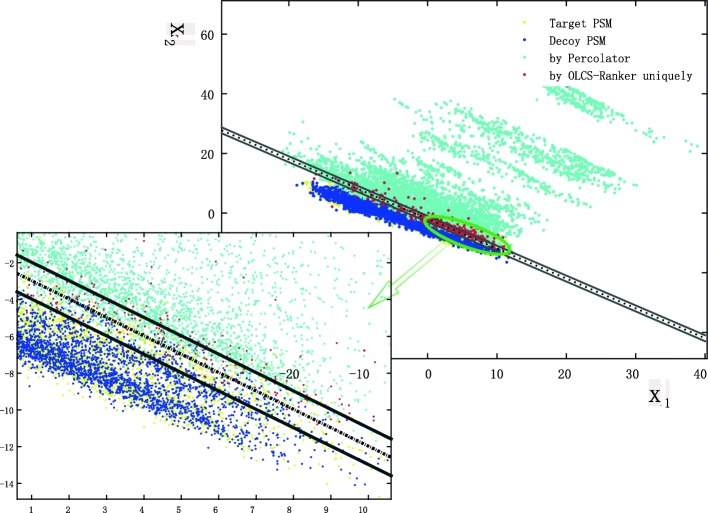
Distribution of identified PSMs by Percolator and OLCS-Ranker. The blue and yellow dots represent target and decoy PSMs, respectively, the cyan dots represent the target PSMs identified by Percolator (98.8% of them have also been identified by OLCS-Ranker), and the red dots represent the target PSMs identified by OLCS-Ranker only. The dotted line represents the linear classifier given by Percolator, and its margin region is defined by the region bounded by the two solid lines. The two-step projection is given as follows. Step 1. Rotate the sample space. Let 〈*b*,*u*〉+*b*_0_=0 be the discriminant hyperplane trained by Percolator, with feature coefficients *b*=[*b*_1_,⋯,*b*_*q*_], intercept *b*_0_, and number of features *q*. Let *P*∈*R*^*q*×*q*^ be orthogonal rotation matrix with *w*=[1,1,0,⋯,0]∈*R*^*q*^ such that *Pw*=*b*. Then the hyperplane after rotation is $\langle P w,u \rangle + b_{0} = 0 \quad \Leftrightarrow \quad \langle w,P_{}^{T} u \rangle + b_{0} = 0 \quad \Leftrightarrow \quad \langle [1,\ 1], [x_{1},x_{2}] \rangle + b_{0} = 0 $, with $ P_{}^{T} u = [x_{1},\cdots, x_{q}]$. PSM *u* in sample space *R*^*q*^ is rotated as $ P_{}^{T} u = [x_{1},\cdots, x_{q}]$. Step 2. Project the rotated PSMs to a plane with the first two rotated coordinates *x*_1_ and *x*_2_ (two axes in the figure). The dotted line 〈[1, 1],[*x*_1_,*x*_2_]〉+*b*_0_=0 is the linear classifier. 〈[1, 1],[*x*_1_,*x*_2_]〉+*b*_0_=+1 and 〈[1, 1],[*x*_1_,*x*_2_]〉+*b*_0_=−1 are the boundaries of the margin of the linear classifier

Not surprisingly, OLCS-Ranker validated more PSMs than other methods in most cases. For a closer look, we compared the outputs by OLCS-Ranker and Percolator on Velos-nomips in Fig. 2. For visualization, we project PSMs in nine-dimensional sample space to a plane which can be seen, as shown in Fig. 2. As we can see, the red dots are mainly distributed in the margin region, and they are mixed with decoy and other target PSMs. Percolator misclassified these red dots, OLCS-Ranker, however, has correctly identified them using nonlinear kernel. Similarly, we have observed this advantage of OLCS-Ranker on Yeast, Tal08 and Velos-mips datasets as well. These figures could be found in Additional file 1.

**Hard datasets and normal datasets**

Note that in Table 2, all the three approaches reported relatively low ratios of validated PSMs on Yeast, Ups1 and Tal08 dataset. As aforementioned, we call them “hard datasets”, in which a large proportion of incorrect PSMs usually increases the complexity of identification for any approach. Particularly, the ratios on Yeast, Ups1 and Tal08 are 0.204 ∼0.219, 0.05 ∼0.062, and 0.096 ∼0.117, respectively, while the ratios on the other datasets (“normal datasets”) are larger than 0.35.

### Model evaluation

**Fig. 3:**
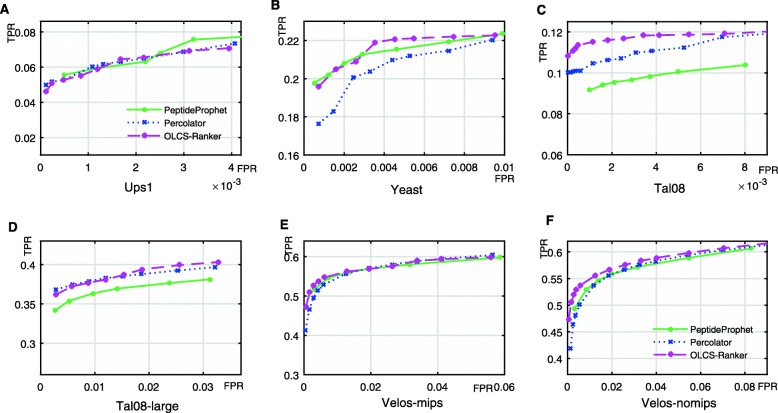
ROC curves. Relationship of TPR and FPR of the identified PSMs by PeptideProphet, Percolator and OLCS-Ranker. **a**. On Ups1; **b**. On Yeast; **c**. On Tal08; **d**. On Tal08-large; **e**. On Velos-mips; **f**. On Velos-nomips

We used receiver operating characteristic (ROC) to compare the performances of OLCS-Ranker, PeptideProphet and Percolator. As shown in Fig. 3, OLCS-Ranker reached highest TPRs among the three methods at most values of FPRs on all datasets. Compared with PeptideProphet, OLCS-CRanker reached significantly higher TPR levels on Tal08 and Tal08-large dataset. Compared with Percolator, OLCS-CRanker reached significantly higher TPR levels on Yeast, Tal08 and Velos-nomips dataset. On Velos-nomips, the TPR values of OLCS-Ranker were about 0.04 higher (i.e., about 8% more identified target PSMs) than that of Percolator with FPR levels from 0 to 0.02 (corresponding FDR levels from 0 to 0.07). In general, OLCS-Ranker outperformed PeptideProphet and Percolator in terms of the ROC curve.

**Fig. 4:**
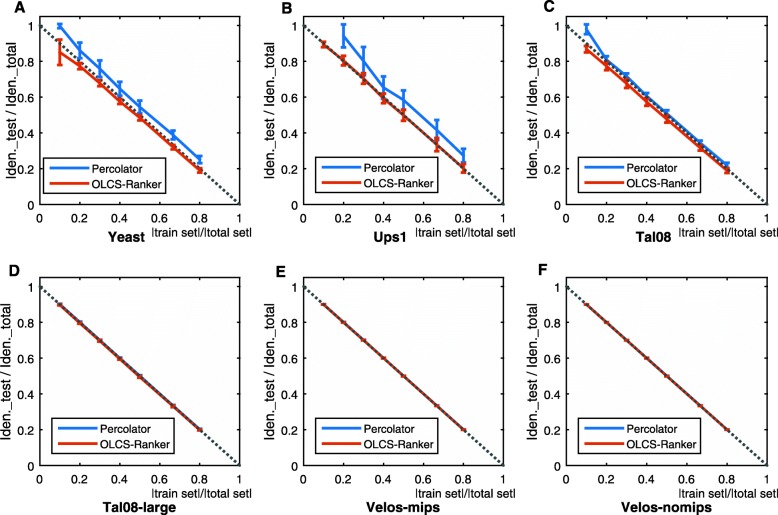
Identified_test/Identified_total versus |train set |/ |total set |. *x*-axis: train/total ratio, the ratio of the number of selected training PSMs to the total number of PSMs in the dataset; *y*-axis: test/total ratio, the ratio of the number of PSMs identified on the test set to the number of PSMs identified in the total dataset. The dotted line segment between (0,1) and (1,0) indicates the expected test/total ratios. **a**. On Yeast; **b**. On Ups1; **c**. On Tal08; **d**. On Tal08-large; **e**. On Velos-mips; **f**. On Velos-nomips

We have also examined model overfitting by the ratio of identified PSMs in the test set to the number of the total identified PSMs (identified_test/identified_total) versus the ratio of the size of training set to the size of total dataset (|train set | / |total set |). As PeptideProphet does not use the supervised learning framework, we only compared OLCS-Ranker with Percolator and CRanker in this experiment. Assume that correct PSMs are identically distributed over the whole dataset. If neither underfitting nor overfitting occurs, then the ratio of identified_test/identified_total should be close to 1 - |train set |/ |total set |. For example, at |train set |/ |total set | =0.2, the expected ratio of identified_test/identified_total is 0.8. Particularly, the training sets and test sets were formed by randomly selecting PSMs from the original datasets according to the values of =0.1,0.2,⋯,0.8. For each value of train/total, we computed the mean value and the standard deviation of the ratios of identified_test/identified_total based on 30 times of running Percolator and OLCS-Ranker, and results were shown in Fig. 4. As we can see, the identified_test/identified_total ratios reported by OLCS-Ranker are closer to the expected ratios than those of Percolator does on Yeast on Ups1. Take |train set |/ |total set | = 0.2 in Fig. 4a, as an example, in which 20%/80% of PSMs were used for training/testing, and the corresponding expected identified_test/identified_total ratio is 0.8. The actual identified_test/identified_total ratio of OLCS-Ranker is 0.773 with standard error 0.018, and 0.861 with standard error 0.043 by Percolator.

Due to the extraordinary running time of CRanker, we only compared OLCS-Ranker and CRanker at |train set |/ |total set | =2/3, and listed the results in Table 3. Although CRanker showed the same ratios of identified_test/identified_total on normal datasets as OLCS-Ranker did, its ratios on hard dataset are less than the expected ratio, 1/3. While the identified_test/identified_total ratio of CRanker is 0.272 and 0.306 on Ups1 and Tal08 respectively, the ratio of OLCS-Ranker is 0.334 and 0.342, respectively. The results indicate that compared with CRanker, OLCS-Ranker overcomes the overfitting problem on hard datasets.

**Table 3:** Comparing OLCS-Ranker with CRanker algorithm

Dataset	Method	#PSMs	$\frac {test}{total} $	RAM (Mb)	time (s)
Yeast	CRanker	1386	0.339	1503.6	667.8
	OLCS-Ranker	1387	0.320	87.2	16.9
Ups1	CRanker	510	0.272	2034.0	1507.0
	OLCS-Ranker	477	0.334	160.2	19.3
Tal08	CRanker	1030	0.306	2347.9	1579.6
	OLCS-Ranker	1150	0.342	28.9	26.0
Tal08-large	CRanker	15531	0.334	6107.9	10090.1
	OLCS-Ranker	15863	0.331	601.0	116.7
Velos-mips	CRanker	117301	0.334	6123.1	9052.9
	OLCS-Ranker	118266	0.333	699.3	495.5
Velos-nomips	CRanker	170092	0.332	6128.9	11478.5
	OLCS-Ranker	172445	0.333	395.7	754.3

Furthermore, we have compared the outputs of Percolator and OLCS-Ranker with different training sets to examine the stability of OLCS-Ranker. Usually, the output of a stable algorithm does not change dramatically along with input training data samples. We have run Percolator and OLCS-Ranker 30 times at each value of |train set |/ |total set | ratio =0.1,0.2,0.3,⋯,0.8.

**Fig. 5:**
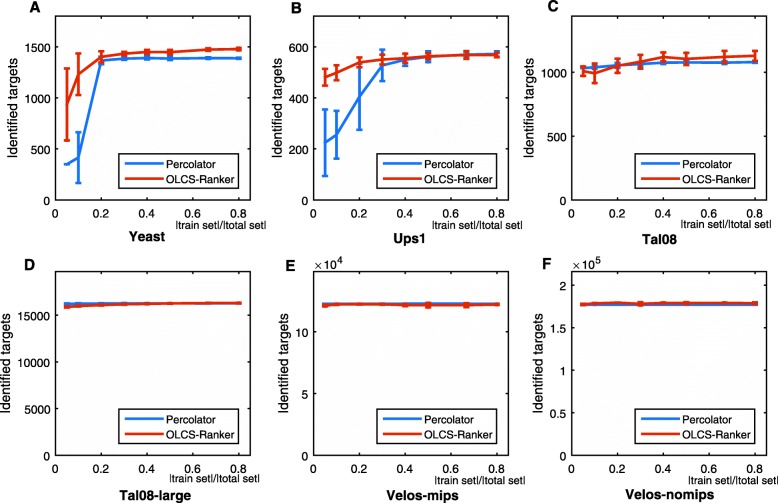
The number of identified target PSMs with various number of training PSMs. *x*-axis: train/total ratio, the ratio of the number of training PSMs to the total number of PSMs the total dataset; *y*-axis: identified targets, the number of identified target PSMs. **a**. On Yeast; **b**. On Ups1; **c**. On Tal08; **d**. On Tal08-large; **e**. On Velos-mips; **f**. On Velos-nomips

The average numbers of identified PSMs and its standard deviations were plotted in Fig. 5. As we can see, both algorithms are stable on normal datasets. However, on Yeast and Ups1, deviations of outputs by OLCS-Ranker are smaller, especially when |train set |/ |total set | ratio is small.

### The algorithm efficiency

In order to evaluate the computational resources consumed by OLCS-Ranker, we compared its running time and used memory with that used by the kernel-based baseline method, CRanker. As the whole training data is needed for CRanker to construct its kernel matrix, it is very time-consuming on large datasets. Instead, CRanker divided the training set into five subsets by randomly selecting 16000 PSMs for each subset. The final score of a PSM is the average of the scores on the five subsets.

Table 3 summarized the comparison of OLCS-Ranker and CRanker in terms of the total number of identified PSMs, the ratio of identified PSMs in the test set to the number of total identified PSMs, used RAM and elapsed time. As we can see, it took CRanker from about 10 min to half an hour on three small datasets, Ups1, Yeast and Tal08, and about 3 h on comparatively large datasets, Tal08-large, Velos-mips and Velos-nomips. In contrast, it took OLCS-Ranker only 13 min on the largest dataset Velos-nomips, about 15∼85 times faster than CRanker. Moreover, OLCS-Ranker consumed only about 1/10 of RAM that used by CRanker on small datasets. On large datasets, OLCS-Ranker has low memory cost. It uses about 400Mb RAM on the tested largest dataset, Velos-nomips. By contrast, CRanker could not efficiently deal with large-scale datasets since large kernel matrix could not load into to memory. The memory of CRanker list in the table is used for training its five small-sized sub-models.

In summary, OLCS-Ranker requires less computational time and memory than C-Ranker does. The analysis is given as follows. CRanker uses a batch learning method in training process and has to maintain a *n*-by-*n* dense kernel matrix, where *n* is the number of PSMs. In contrast, OLCS-Ranker uses an online learning algorithm, which iteratively trains the model by taking only one data sample at each round. Moreover, OLCS-Ranker only needs to keep data samples in the active set in the memory. Hence, the requirement of computational resources during the model-training process is significantly reduced.

Particularly, the memory required by CRanker is *O*(*n*^2^), with *n* the number of training PSMs, while it is *O*(|*S*|^2^) required by OLCS-Ranker, where |*S*| is the number of PSMs in the active set *S*. As the value of *n* is usually very large, CRanker can hardly run a dataset with more than 20,000 PSMs on a normal PC. However, the maximum size of the active set |*S*| in OLCS-Ranker is pre-selected and far less than the value of *n* for large datasets.

From the perspective of computational complexity, CRanker needs to solve a series of convex sub-problem. Each subproblem is essentially an SVM classification problem, and the computational complexity is between *O*(*n*^2^) and *O*(*n*^3^). Thus, the computational complexity of CRanker is at least *O*(*n*^2^). However, OLCS-Ranker deals with one PSM sample, at the computational cost of *O*(|*S*|^2^), at each round. Thus, the computational complexity of OLCS-Ranker is bounded by *O*(*n*|*S*|^2^), which is usually far less than that of CRanker when |*S*|≪*n*.

### Evaluation by the entrapment sequence method

The entrapment sequence method was introduced as an alternative of target-decoy strategy to validate true PSMs in mass spectrometry data analysis. We have evaluated the performance of OLCS-ranker with the entrapment sequences obtained from “Pfu” dataset published in reference [20].

**Fig. 6:**
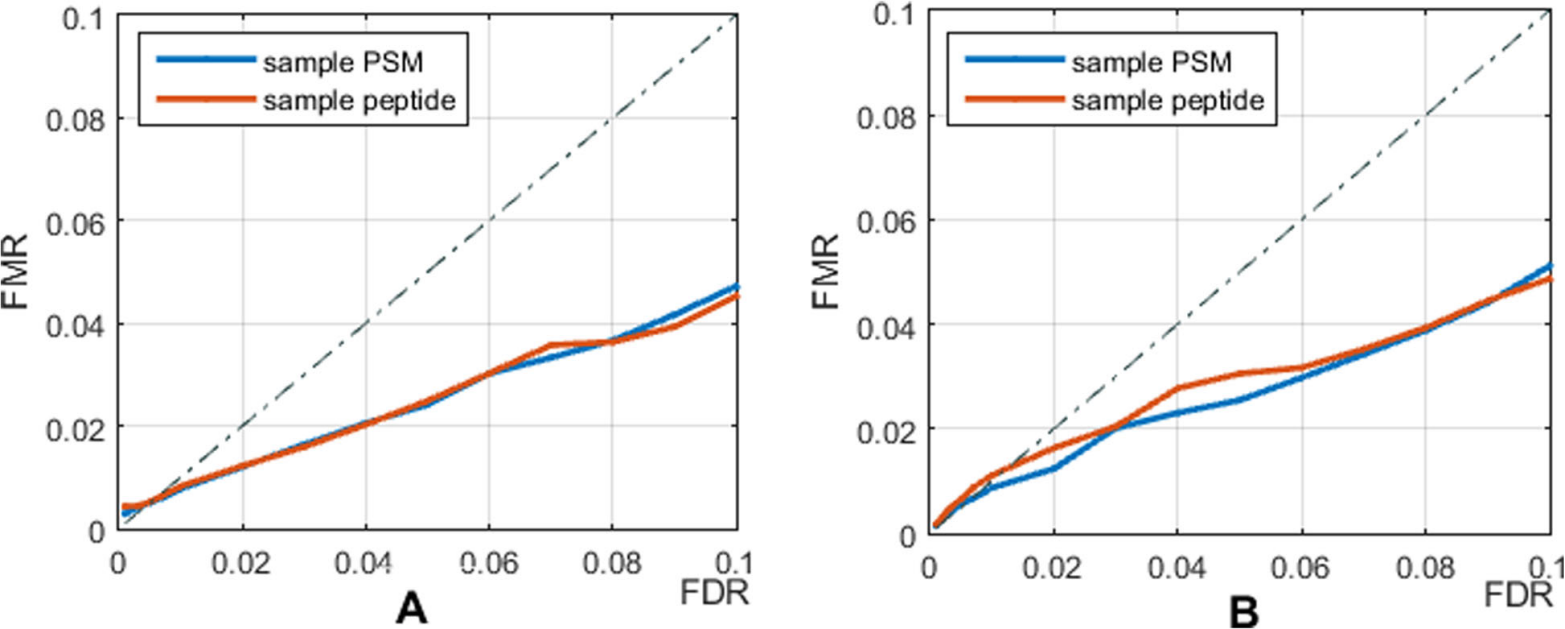
False match rate under various FDR level on Pfu dataset of OLCS-Ranker, FMR =$N_{\text {entrapment}} / N_{\text {target}}^{}$. **a**. Tide + OLCS-Ranker; **b**. Comet + OLCS-Ranker

We use the entrapment hits to calculate the false match rate (FMR) to assess the quality of the identification results. Fig. 6 depicts corresponding FMRs under a series of FDR levels of OLCS-Ranker. It is shown that with both Tide (Fig. 6a) and Comet (Fig. 6b) search engines, OLCS-Ranker has approximately lower FMR levels than those of FDRs in identified sample PSMs and peptides, which indicates the identification results are reasonable according to the definition of FMR.

We also compared the identification results of OLCS-Ranker using different search engines with those in [20] under 0.01 FDR for PSM and peptide, respectively, and results are listed in Table 4. It is shown that in most cases the FMRs estimated by entrapment hits are roughly equal to 0.01. Particularly, with the Comet search engine at FMR =0.009, OLCS-Ranker identified 10603 PSMs, 6% more than those identified by Crux Percolator. Similarly for identified peptides, the number given by OLCS-Ranker is about 6% (5667−5343)/5343=6.06*%*) more than that of Crux Percolator. With the Tide search engine, OLCS-Ranker identifies approximately the same number of PSMs and peptides as those of Crux Percolator, but has lower FMR levels. Thus, in terms of identification number and FMRs given by this entrapment sequence test, OLCS-Ranker has shown the quality of its identified results is at least as high as that of Crux Percolator.

**Table 4:** The identification numbers and FMRs under FDR =0.01 on Pfu dataset of various searching methods

	Method	Identification number	FMR
Sample PSM	Tide + Crux percolator	6799	0.013
	X!Tandem + percolator	9889	0.011
	Mascot + PepDistiller	9864	0.013
	Comet + Crux Percolator	9922	0.009
	Tide + OLCS-Ranker	6897	0.008
	Comet + OLCS-Ranker	10603	0.009
Sample peptide	Tide + crux percolator	3878	0.016
	X!Tandem + percolator	5320	0.012
	Mascot + PepDistiller	5360	0.015
	Comet + crux percolator	5343	0.010
	Tide + OLCS-ranker	3806	0.008
	Comet + OLCS-ranker	5667	0.011

## Conclusions

We have presented a cost-sensitive post-database search approach, OLCS-Ranker, for peptide identification to overcome the challenges of “hard datasets” and scalability problem with the kernel-based learning model. We designed an online cost-sensitive model to tackle a large portion of decoy PSMs in hard datasets by assigning them larger penalties. Moreover, OLCS-Ranker has shown better scalability than CRanker due to significantly reduced memory requirement and total training time. Experimental studies have shown that OLCS-Ranker outperformed benchmark methods in terms of accuracy and stability. Also, compared with CRanker, OLCS-Ranker is about 15 ∼85 times faster over tested datasets and has overcome the overfitting problem on hard datasets.

## Materials and methods

### Basic CRanker model

CRanker [14] cast identification of target PSM as a classification problem. Let $\Omega =\{x_{i}^{},y_{i}^{}\}_{i=1}^{n} \subseteq R_{}^{q} \times \{-1,1\}$ be a set of *n* PSMs, where $x_{i}^{} \in R_{}^{q}$ represents its *i*-th PSM record with *q* attributes, and $y_{i}^{} \in \{ 1, -1\}$ is the corresponding label indicating a target or decoy PSM. Define $ \Omega _{+}^{} = \{j \,|\, y_{j}^{} = 1 \}, \quad \Omega _{-}^{} = \{j \,|\, y_{j}^{} = -1 \}. $ The identification task is to train a discriminant function for filtering out the correct PSMs from the target PSMs (ones with labels “ +1”).

While class labels in a standard classification problem are all trustworthy, a large number of “ +1” labels in PSM identification are not correct. CRanker [14] introduced weight *θ*_*i*_∈[0,1] for each PSM sample (*x*_*i*_,*y*_*i*_) to indicate the degree of the reliability of the label *y*_*i*_. Particularly, *θ*_*i*_=1 indicates that label *y*_*i*_ is definitely correct, *θ*_*i*_=0 indicates that it is definitely incorrect, and *θ*_*i*_∈(0,1) indicates that label *y*_*i*_ is probably correct. In fact, all “ −1” labels (decoy PSMs) are correct, and thus *θ*_*i*_=1 for all $i\in \Omega _{-}^{}$. Based on Support Vector Machine (SVM) [27], CRanker can be solved by the following optimization problem

1 $$ \begin{array}{cl} \min_{w,\theta}^{} & \frac{1}{2}\|w\|^{2} + C \sum_{i=1}^{n} \theta_{i}^{} h(y_{i}^{}f(x_{i})) - \lambda \sum_{i=1}^{n} \theta_{i}^{} \\ \text{s.\,t.} & \theta_{i}^{} = 1, \ i \in \Omega_{-}^{}, \\ & 0\leq \theta_{i}^{} \leq 1,\ i \in \Omega_{+}^{}, \\ \end{array}  $$

where *C*>0 is the regularization parameter, *λ*>0 is the parameter controlling the number of identified PSMs, *h*(*t*)= max(0,1−*t*) is the hinge loss function, and *f*(*x*_*i*_)=〈*w*,*ϕ*(*x*_*i*_)〉 is the value of discriminant function at *x*_*i*_ with feature mapping *ϕ*(·). As shown in [28, 29], a larger value of parameter *λ* selects more PSMs into the training process.

### Cost-sensitive ranker model

In this section, we present a cost-sensitive (CS) classification model to partially tackle the stability problem of CRanker over datasets with a distribution of unbalanced PSMs. Unlike the CRanker model, the CS model uses different loss functions for decoy and target PSMs. In fact, learning errors should be treated with different penalties in peptide identification. If the discriminant function assigns “ +1” label to a decoy PSM, then we know for sure that the label assignment is wrong. In this case, the learning error is more likely caused by the model itself rather than the quality of the data sample, and hence we should give the loss function a large penalty. On the other hand, if a target is classified as negative and assigned label “ −1”, we are not even sure whether the label assignment is correct, and thus we consider a small penalty for the loss function. Based on these observations, we incorporate the new penalty policy into model (1) and the new model is described as follows:

2 $$ \begin{array}{cl} \min_{w,\theta}^{} & \frac{1}{2}\|w\|^{2} + C_{1} \sum_{i\in\Omega_{-}^{}}^{} \theta_{i}^{} h(y_{i}^{}f(x_{i}))\\ & \quad + C_{2} \sum_{i\in\Omega_{+}^{}}^{} \theta_{i}^{} h(y_{i}^{}f(x_{i})) - \lambda \sum_{i=1}^{n} \theta_{i}^{} \\ \text{s.\,t.} & \theta_{i}^{} = 1, \ i \in \Omega_{-}^{}, \\ & 0\leq \theta_{i}^{} \leq 1,\ i \in \Omega_{+}^{}, \\ \end{array}  $$

where *C*_1_>0, *C*_2_>0 are weights for the losses of the decoys and targets, respectively. Model (2) is named **cost-sensitive ranker** model and denoted by **CS-Ranker**. As we choose a larger penalty for decoy losses, $C_{1}^{} \geq C_{2}^{}$ always holds.

### The convex-concave procedure for solving CS-Ranker

In order to solve the CS-Ranker model, we transform (2) to its DC (**d**ifference of two **c**onvex functions) form. According to the method in [29], if a pair of *w*^∗^∈*R*^*n*^ and *θ*^∗^∈*R*^*n*^ is an optimal solution to CS-Ranker model (2), then *w*^∗^ is also an optimal solution of the following problem

3 $$ \min_{w}^{} \, \frac{1}{2}\|w\|^{2} + C_{1} \sum_{i\in\Omega_{-}^{}}^{} h(y_{i}^{}f(x_{i})) + C_{2} \sum_{i\in\Omega_{+}^{}}^{} R_{s}^{}(y_{i}^{}f(x_{i}))  $$

where *R*_*s*_(*t*)= min(1−*s*, max(0,1−*t*)), $s = 1- \frac {\lambda }{C_{2}^{}}$.

Since *R*_*s*_(*t*)=*H*_1_(*t*)−*H*_*s*_(*t*), with *H*_*s*_(*t*)= max(0,*s*−*t*) and *H*_1_(*t*)= max(0,1−*t*), then model (3) can be recast as

4 $$ \min \quad J(w) = J_{\text{vex}}^{}(w) + J_{\text{cav}}^{}(w),  $$

where

5 $$ \begin{array}{l} J_{\text{vex}}^{}(w)= \frac{1}{2}\|w\|^{2} + C_{1}^{} \sum_{i\in\Omega_{-}^{}}^{} H_{1}(y_{i}^{}f(x_{i})) \\ \qquad + C_{2}^{} \sum_{i\in\Omega_{+}^{}}^{} H_{1}(y_{i}^{}f(x_{i})), \\ J_{\text{cav}}^{}(w) = - C_{2}^{} \sum_{i\in\Omega_{+}^{}}^{} H_{s}^{}(y_{i}^{}f(x_{i})). \end{array}  $$

$J_{\text {vex}}^{}(\cdot)$ and $J_{\text {cav}}^{}(\cdot)$ are convex and concave functions respectively. Hence, Problem (4) can be solved by a standard Concave-Convex Procedure (CCCP) [30], which iteratively solves subproblems

6 $$ w_{}^{k+1} = \text{argmin}_{w}^{} \quad J_{\text{vex}}^{}(w) + J_{\text{cav}}^{\prime}(w^{k})\cdot w  $$

with initial *w*^0^. The subproblem (6) can be solved by its Lagrange dual [31]:

7 $$ {{}\begin{aligned} \begin{array}{cl} \max_{\alpha}^{} & G(\alpha) \,=\, -\frac{1}{2}\sum_{i,j}^{}\alpha_{i}^{} \alpha_{j}^{} k(x_{i},x_{j}) + \langle \alpha,y \rangle + \sum_{i\in \Omega_{+}}^{} C_{2} \eta_{i}^{k} \\ \text{s.\,t.} & A_{i} \leq \alpha_{i} \leq B_{i}, \quad, i=1,\ldots, n \\ & A_{i} = \min(0,C_{1}^{} y_{i}),\ i\in \Omega_{-}^{} \\ & B_{i} = \max(0,C_{1}^{} y_{i}),\ i\in \Omega_{-}^{} \\ & A_{i} = \min(0,C_{2}^{}y_{i})-C_{2}^{}\eta_{i}^{}y_{i},\ i\in \Omega_{+}^{} \\ & B_{i} = \max(0,C_{2}^{}y_{i})-C_{2}^{}\eta_{i}^{}y_{i},\ i\in \Omega_{+}^{} \\ \end{array} \end{aligned}}  $$

where $\eta _{i} = \left \{ \begin {array}{cl} 1, & \text { if}\ y_{i} f^{}(x_{i}) < s,\\ 0, & \text { otherwise }. \end {array} \right.$

Model (7) is a kernel-based learning model with *k*(·,·) the kernel function. Then *k*(*x*_*i*_,*x*_*j*_) calculates, in feature space, the pairwise inner product of PSM records of *x*_*i*_ and *x*_*j*_, which are represented in vector format. Hence, OLCS-Ranker can handle PSM records generated by any search engine as long as the output PSMs are represented in vector format.

### The online learning algorithm for CS-Ranker model

Inspired by the work in [32, 33], we obtain the discriminant function for CS-Ranker by solving its DC form (3).

Different from classical classifiers which take all PSM samples at once, the **online CS-Ranker algorithm** (**OLCS-Ranker**) iteratively trains the discrimination function and adds only one PSM sample into the training process at each iteration. The PSM sample is randomly selected to prevent the solution of (3) from trapping at a local minimum and its effectiveness has been observed in approaches such as stochastic gradient descent [34]. In order to reduce the cost of memory and computation, OLCS-Ranker maintains an active set which keeps only indices of PSMs that determine the discriminant function in model training, and the PSMs that do not affect the discriminant function are discarded.

#### Online algorithm for solving CS-Ranker

The implementation of OLCS-Ranker is depicted in Algorithm 1. Particularly, given a chosen PSM sample (Line 3), OLCS-Ranker updates bounds *A*_*j*_, *B*_*j*_, for all $j \in \Omega _{+}^{}\cap S$ (Line 4 – Line 7), and calls subroutines PROCESS and REPROCESS to solve dual programming (7) with training samples in active set *S* (Line 8–Line 12). Iteratively, the algorithm calls subroutine CLEAN to remove part of redundant PSMs from the active set (Line 13). The iteration terminates when all the training PSMs have been chosen for training.


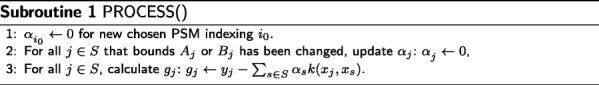


#### Subroutines

Subroutine PROCESS ensures that all the coordinates of *α*_*j*_ satisfy the bound constraint conditions in CS-Ranker model (7). It initializes $\alpha _{i_{0}}$ with 0, where *i*_0_ is the index of the chosen PSM, and updates the coordinates *α*_*j*_ if bound *A*_*j*_ or *B*_*j*_ has changed (Line 1-2). Then, it updates gradient vector *g*_*j*_, *j*∈*S* (Line 3), where *g* is defined by

8 $$ g_{i} \stackrel{\triangle}{=} \frac{\partial G(\alpha)}{\partial \alpha_{i}} = y_{i} - \sum_{j\in S}^{} \alpha_{j}^{}k(x_{i},x_{j}).  $$

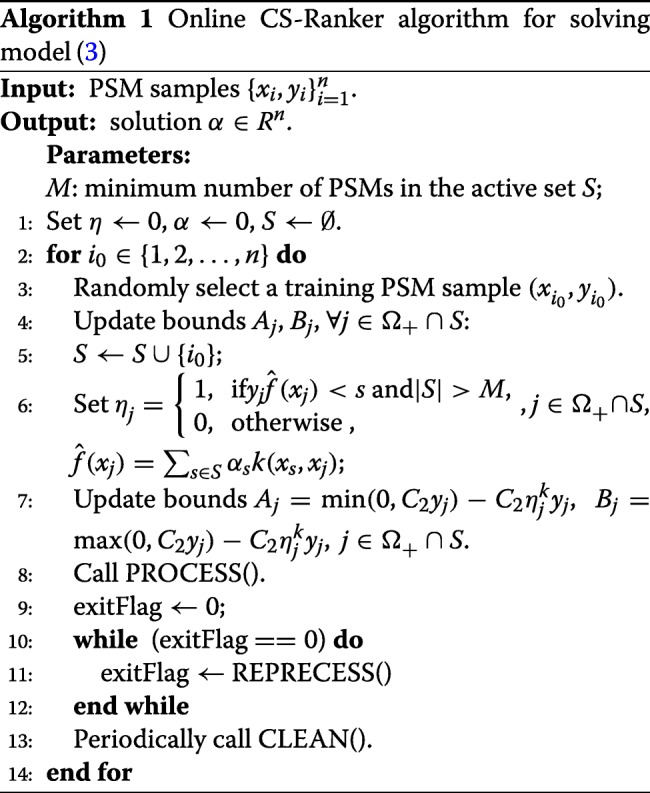


Subroutine REPROCESS aims to find a better solution of model (7). It selects the instances with the maximal gradient in active set *S* (Line 1 – Line 12). Once an instance is selected, it computes a stepsize (Line 13 – Line 17) and performs a direction search (Line 18 – Line 19). The derivation of these iteration formulae could be found in Additional file 1.


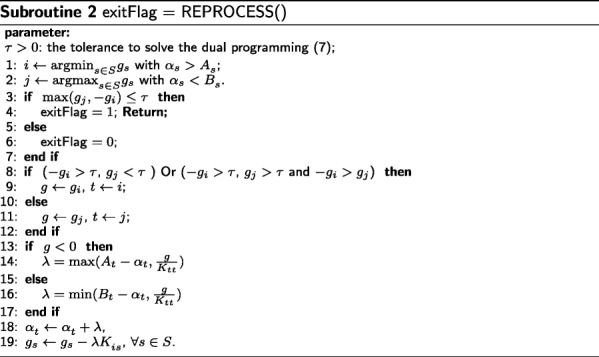


Subroutine CLEAN removes PSMs that are not effective to the discriminant function from the active set *S* to minimize the requirement of memory and computation. The subroutine selects non-support vectors and keeps them in set *V* (Line 1 – Line 4), then selects at most *m* PSMs of *V* with the largest gradients, and finally removes them from *S* (Line 5 – Line 9).


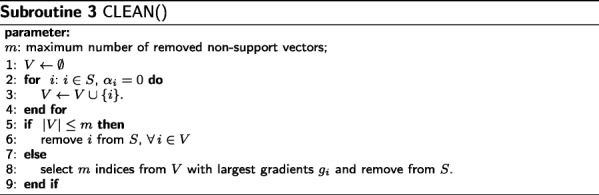


#### Calculate PSM scores

After discriminant function $\hat {f}$: $ \hat {f}(x) = \sum _{j\in S}^{} \alpha _{j} k(x_{j},x),$ where *k*(·) is the selected kernel function, is trained, we calculate the scores of all PSMs on both training and test sets. The score of PSM (*x*_*i*_,*y*_*i*_) is defined in [14]:

$$score(i) = \frac{2}{\pi}\arctan(\hat{f}(x_{i})). $$

The larger the score value is, the more likely a PSM is correct. The PSMs are ordered according to their scores, and a certain number of PSMs are reported according to a pre-selected FDR.

## Supplementary information

**Additional file 1** Additional results. The derivation of iteration formulae of OLCS-Ranker and some additional results.

**Additional file 2** Cross validation results. Details of the Cross validation results (Excel file).

## Data Availability

The datasets supporting the conclusions of this article are available in the Figshare repository, 10.6084/m9.figshare.5739705.v1. The software of OLCS-Ranker can be download from https://github.com/Isaac-QiXing/CRanker. A web-based GUI for users of OLCS-Ranker is provided at http://161.6.5.181:8000/olcs-ranker/.

## Abbreviations

MS/MS: Tandem mass spectrometry
PSM: Peptide-spectrum match
BNP: Bayesian nonparametric model
SVM: Support vector machine
TPP: Trans-Proteomic Pipeline
CS-Ranker: Cost-sensitive Ranker
OLCS-Ranker: Online algorithm for CS-Ranker
Ups1: Universal proteomics standard set
Yeast: S.cerevisiae Gcn4 affinity-purified complex
PBMC: Human Peripheral Blood Mononuclear Cells
Velos-mips: LTQ-Orbitrap Velos with MiPS
Velos-nomips: LTQ-Orbitrap Velos with MiPS-off
ROC: Receiver operating characteristic
DC: Difference of two convex functions
CCCP: Concave-Convex Procedure
FMR: False match rate
