# Supplementary material for: A cost-sensitive online learning method for peptide identification
Source: BMC Genomics. 2020 Apr 25;21:324. doi: 10.1186/s12864-020-6693-y (PMC7183122; doi:10.1186/s12864-020-6693-y)
Supplement: Supplementary file 1 — Additional file 1 Additional results. The derivation of iteration formulae of OLCS-Ranker and some additional results. [file 12864_2020_6693_MOESM1_ESM.pdf]

Supporting Information

The rule for choosing candidate samples in CLEAN subroutine

The rule for removing candidate indices in CLEAN subroutine is based on the relationship between gradient vector g and the predicted discriminant function value $\hat{f}(x)$. We have the following observation:

- Given $g = \nabla W(\alpha) = y - K\alpha$, $g_i = y_i - K_i^T\alpha = y_i(1 - y_i\hat{f}(x_i))$.
- If $y_i = -1$, then $g_i = -1 - \hat{f}(x_i)$, $\alpha_i \in [-c_1, 0]$. When $\hat{f}(x_i) \ll -1 \ \Leftrightarrow \ g_i \gg 0$, $\{x_i, y_i\}$ is separated correctly, and we can safely remove index i from the active set.
- If $y_i = +1$, then $g_i = 1 - \hat{f}(x_i)$.
  - If $\hat{f}(x_i) \gg 1$, then $\alpha_i \in [0, c_2]$, $g_i = 1 - \hat{f}(x_i) \ll 0$. In this case, $\{x_i, y_i\}$ is separated correctly, and we can safely remove index i from the active set.
  - If $\hat{f}(x_i) \ll s$, then $\alpha_i \in [-c_2, 0]$, $g_i = 1 - \hat{f}(x_i) \gg 1 - s > 0$. In this case, $\{x_i, y_i\}$ is safely identified as an incorrect target PSM, we can remove index i from the active set.

Hence, the rule for removing candidate indices is defined as follows.

Rule 1. $\forall\, i$ with $\alpha_i = 0$, $i \in S$:

If $y_i = -1$, $g_i \geq \mu_{\text{safe}}$ OR $y_i = +1$, $g_i \geq 1 - s + \mu_{\text{safe}}$, OR $y_i = +1$, $g_i \leq -\mu_{\text{safe-target}}$, then

$$V \leftarrow V \cup i$$

Note that if we set $\mu_{\text{safe-target}} = +\infty$, then all the identified correct target PSMs will not be removed from S and remain in the active set.

Effects of model parameter C_2 and algorithm parameters

The identified numbers of PSMs with different settings of the algorithm parameters τ and μ_{safe} are listed in Table S1. As there is no significant difference with the identified numbers of PSMs, we used the same setting of $\tau = 0.05$ and $\mu_{\text{safe}} = 0.3$ on all datasets in the experimental study.

Distribution of identified PSMs by Percolator and OLCS-Ranker on each datasets

CLEAN

parameter:

m: maximum number of removed non-support vectors;

μ_{safe}, $\mu_{\text{safe-target}}$: two thresholds to select candidate PSMs.

$V \leftarrow \emptyset$
for i: $i \in S$, $\alpha_i = 0$ **do**
  if $y_i = -1$, $g_i \geq \mu_{\text{safe}}$ OR $y_i = +1$, $g_i \geq 1 - s + \mu_{\text{safe}}$ OR $y_i = +1$, $g_i \leq -\mu_{\text{safe-target}}$ **then**
    $V \leftarrow V \cup \{i\}$.
  end if
end for
if $|V| \leq m$ **then**
  remove i from S, $\forall\, i \in V$
else
  select m indices from V with largest gradients g_i and remove from S.
end if

Table S1: The effects of algorithm parameters

Dataset	TP	time (s)	τ	μ_{safe}	C_1	C_2	λ
Yeast	1465.8	15.83	0.05	0.1	4.8	2.4	2.4
	1466.7	16.16	0.05	0.2	4.8	2.4	2.4
	1471.5	17.12	0.05	0.3	4.8	2.4	2.4
	1473.2	17.91	0.05	0.4	4.8	2.4	2.4
	1473.7	22.50	0.01	0.3	4.8	2.4	2.4
	1474.4	28.61	0.005	0.3	4.8	2.4	2.4
Tal08	1126.2	20.08	0.05	0.1	0.9	0.6	0.6
	1131.1	22.06	0.05	0.2	0.9	0.6	0.6
	1119.9	27.16	0.05	0.3	0.9	0.6	0.6
	1116.5	31.94	0.05	0.4	0.9	0.6	0.6
	1129.0	29.54	0.01	0.3	0.9	0.6	0.6
	1134.7	32.31	0.005	0.3	0.9	0.6	0.6

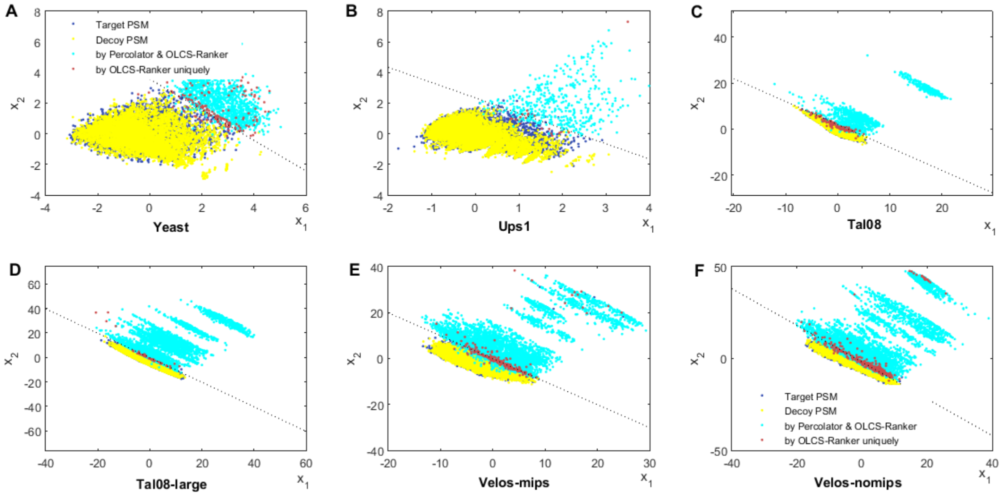

Figure S1: Distribution of identified PSMs by Percolator and OLCS-Ranker. For visualization, the PSMs in nine-dimensional sample space are projected to a proper plane and are shown in the figure. The blue and yellow dots represent target and decoy PSMs, respectively, the cyan dots represent the target PSMs identified by Percolator, and the red dots represent the target PSMs identified by OLCS-Ranker only. The dotted line represents the linear classifier given by Percolator.
